# Development and validation of gallstone disease risk factor identification model: a cross-sectional study in Western China

**DOI:** 10.3389/fnut.2026.1814244

**Published:** 2026-06-23

**Authors:** Xiaofeng Jing, Qiang Zhang, Tiecheng Zhang, Haiqi Xiang, Wenhao Lv, Kai Wen

**Affiliations:** 1 Department of Hospital Infection-Control, West China Hospital Sichuan University Jintang Hospital. Jintang First People’s Hospital, Chengdu, China; 2 Department of Public Health, Chengdu Medical College, Chengdu, China; 3 Department of Big Health and Intelligent Engineering, Chengdu Medical College, Chengdu, China; 4 Department of Digital Orthopaedic Engineering Center, West China Hospital of Sichuan University, Chengdu, China; 5 Department of Medical Affairs, Nanchong Mental Health Center of Sichuan Province, Nanchong, China

**Keywords:** random forest, gallstone, machine learning, risk factor identification model, cross-sectional study

## Abstract

**Background:**

Gallstone disease poses a growing public health concern worldwide, yet approaches for its identification remain unclear.

**Objective:**

This study aimed to establish a risk factor identification model by identifying risk factors for gallstone disease, thereby providing evidence-based support for disease prevention.

**Methods:**

Health examination data and questionnaire data were analyzed. Continuous and categorical variables were compared using t-test or chi-square test. LASSO regression with 10-fold cross-validation was used for variable selection. Logistic regression and random forest models were constructed in parallel. Data were randomly split into training and validation sets (7:3), and 10-fold cross-validation assessed model stability in training set. Model discrimination, calibration, and clinical utility were evaluated using the AUC, calibration curves, and decision curve analysis (DCA).

**Results:**

Among the 17,768 subjects, 1,673 comprised the gallstone case group and 16,095 the control group. A random forest model was constructed and demonstrated superior performance to logistic regression, with an AUC of 0.747 (95% CI: 0.724–0.771), specificity of 0.91, sensitivity of 0.86, Brier score of 0.073 and F1 score of 63.41%. Calibration curves showed good consistency between predicted and observed probabilities. DCA confirmed that the random forest model provided higher net benefit across a wide range of clinically relevant thresholds compared with treat-all or treat-none strategies.

**Conclusion:**

The random forest model demonstrated moderate discriminatory ability and clinical relevance for gallstone disease. Confirmed risk factors include TBA, smoking, diabetes, TG, TP, passive smoking, tea consumption, nutrient intake exercise, etc. Identification and intervention targeting these factors may help reduce the incidence of gallstone disease. But as a cross-sectional study, future prospective cohort studies are needed for external validation to establish a robust prediction model.

## Introduction

Gallstone disease represents one of the most prevalent digestive disorders worldwide (1). It’s primarily resulting from impaired gallbladder emptying and bile stasis (2). Beyond its local biliary manifestations, this condition is increasingly recognized as being associated with systemic metabolic disturbances, which elevate the risk of severe complications such as pancreatitis and gallbladder cancer (3). Recently research indicate that gallstones affect approximately 6.3 million men and 14.2 million women in the United States, with associated healthcare expenditures over $6.2 billion annually (4, 5). Notably, the global incidence of gallstone disease continues to rise, which increase the societal healthcare burden (6).

A major challenge in managing gallstone disease lies in its frequently asymptomatic presentation during early stages, thereby complicating timely detection and intervention (7). Although abdominal ultrasonography serves as the standard diagnostic tool for confirming the presence of gallstones, it is not suitable for population-wide screening, or identifying high-risk individuals who prior to symptom onset (8). Consequently, there remains unclear for reliable strategies to enable risk identification of gallstone in clinical practice.

Therefore, our study based on the health examination cohort study baseline database, which aggregates health examination records from 11 hospitals across western China. By integrating these comprehensive datasets, we aim to develop a risk factor identification model for gallstones. This model is intended to provide the identification of high-risk individuals, to further reduce both the incidence and societal burden of gallstone disease.

## Methods

### Ethic statement

This retrospective research conforms to the ethical guidelines of the Declaration of Helsinki. Participants were informed about the use of their clinical and questionnaire data, the collection of biological samples, the long-term storage of these samples, and the future research purposes. The study was approved by the ethics committee of West China Hospital, Sichuan University (Approval No. 2018-491).

### Participants

The database was integrating health examination data from 11 hospitals in Sichuan Province (9). Participants were included if they were adults aged ≥ 18 years who had undergone a health check-up. Exclusion criteria consisted of incomplete patient data or missing information due to other factors.

Gallstone disease was defined as the presence of one or more echogenic foci with posterior acoustic shadowing within the gallbladder lumen on abdominal ultrasonography.

To ensure comparability of patient information, West China Hospital of Sichuan University assessed the consistency of laboratory instruments, imaging technologies, and data management systems used across the alliance hospitals. Strict data cleaning and quality control were implemented for accuracy (10).

Physical examinations included precise measurement of body mass index (BMI) using an ultrasonic automatic body composition analyzer SG-1000SC (Chioy, China), blood pressure measurements taken from the right arm of seated subjects after a 5-min rest, using a tunnel-type ABP-1000 (Version F) sphygmomanometer (Chioy, China) (9).

All participants were required to fast for 10–12 h prior to blood and urine sample collection. All sample analyses were performed in hospital clinical laboratories following established protocols to ensure measurement consistency.

Participants underwent a comprehensive suite of imaging examinations, including ultrasonography, X-ray, chest CT scans, and electrocardiography. The diagnosis of gallstone disease was based on ultrasonography results obtained using an EPIQ5 system (PHILLIPS, USA) (9).

The health screening questionnaire, developed independently by West China Hospital, contained 60 items for males and 58 items for females, covering demographic characteristics, lifestyle factors, sleep quality assessment, and history of chronic diseases. All questionnaire-based lifestyle and dietary variables were collected as categorical frequency data. The frequency categories were defined as never, occasionally, 1–2 times/week, 3–5 times/week, and >5times/week, with a reference period of the past 12 months.

**Figure 1:**
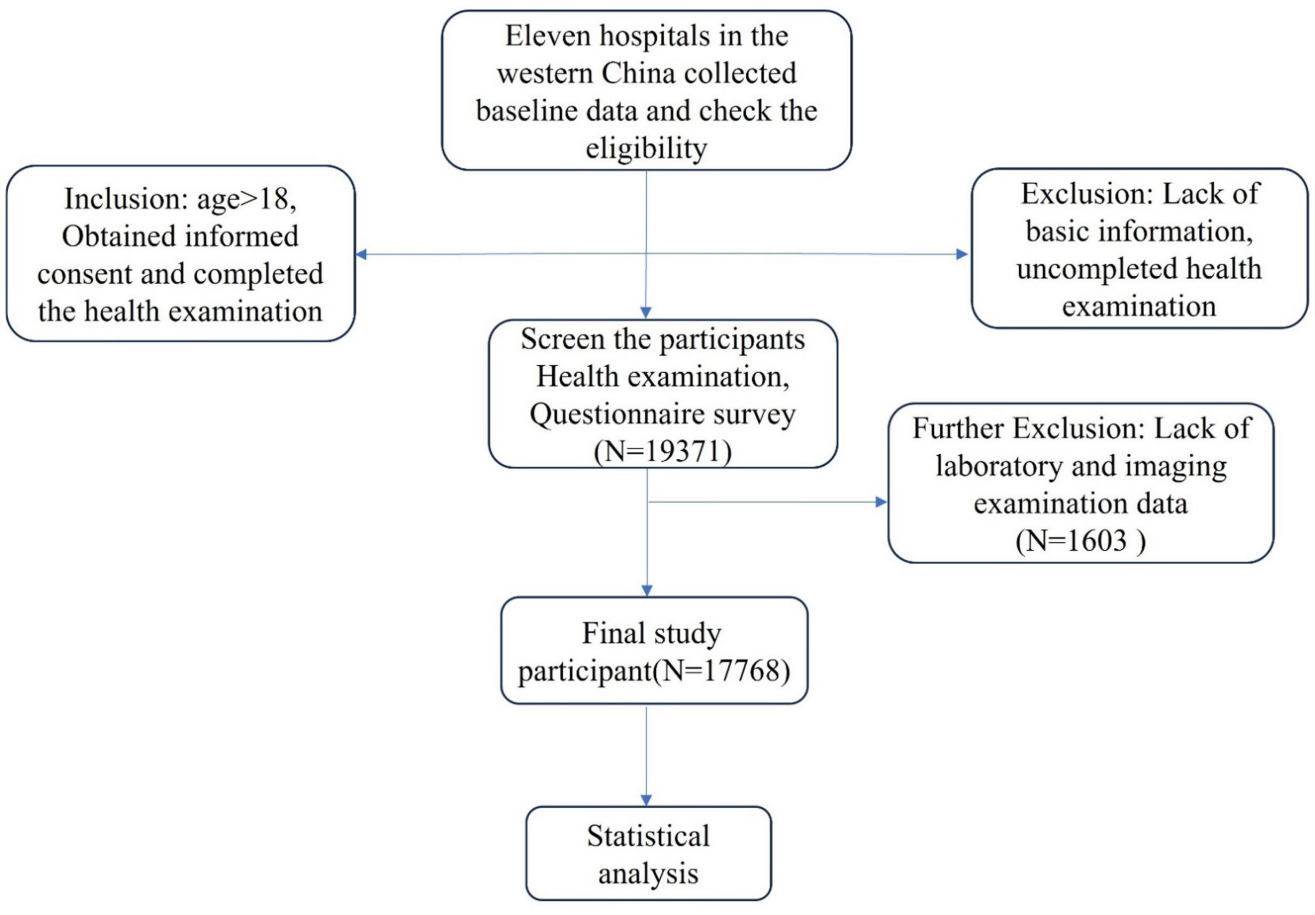
Workflow of the study population selection.

After excluding subjects with incomplete information, a total of 17,768 participants were ultimately included in the study. The participant selection procedure is detailed in Figure 1.

### Study variables

Based on previous studies and the specific context of our research (11, 12), a total of 39 variables were selected for analysis. These variables can be classified as basic demographic character: age, sex, marriage, exercise, smoke, passive smoke, drink; Health conditions: chronic obstructive pulmonary disease (COPD), tumor, diabetes, high blood pressure (HBP), waist, pulse, sleep, BMI. COPD, diabetes, HBP and cancer were based on self-reported previous medical history. For participants who self-reported HBP and/or diabetes, on-site blood pressure measurement and blood glucose testing were performed to verify the conditions; Dietary habits: frequency of consuming coffee, frequency of consuming nutrient, frequency of consuming sweetmeat, frequency fried food consumption (FRY), frequency of consuming tea, drink water, cooking oil, taste, eating speed; Laboratory examinations: low density lipoprotein (LDL), high density lipoprotein (HDL), indirect bilirubin (IBIL), total bile acid (TBA), alanine aminotransferase (ALT), total protein(TP), direct bilirubin (DB), cholesterol(CHO), total bilirubin (TBIL), potassium (POT), aspartate aminotransferase (ASP), triglyceride (TG), albumin, lactic dehydrogenase (LDH) and alkaline phosphatase(ALP).

### Statistical analysis

Data store and management via Microsoft 365. Statistical analysis was performed by using Stata MP 17.0. The Shapiro–Wilk test was priorly used to check the normality of the data distribution. Continuous data were expressed as the mean (standard deviation) and two-tailed Student’s *t*-test was used to compare their difference. Chi-square test and Wilcoxon rank-sum test were used to compared categorical variables between two groups.

Variable selection was performed using the least absolute shrinkage and selection operator (LASSO) regression, the optimal tuning parameter (*λ*) was chosen via 10-fold cross-validation using the one-standard-error rule. Following variable selection by LASSO regression, logistic regression and random forest models were developed to predict gallstone disease. The dataset was randomly split into a training set and a testing set at a 7:3 ratio (hierarchical segmentation). All model development, hyperparameter tuning, and internal validation were performed exclusively on the training set, using 10-fold cross-validation to optimize hyperparameters and assess stability. Final model performance was evaluated on the testing set.

To interpret the random forest model, SHAP (Shapley Additive Explanations) analysis was used. SHAP bar plots ranked predictors by global importance, and SHAP partial dependence plots (PDP) were employed to reveal interactions and their directional effects on gallstone probability.

Discriminative ability was assessed by the area under the receiver operating characteristic curve (ROC) on the testing set. Calibration was evaluated with calibration curves and the Hosmer–Lemeshow test. Decision curve analysis (DCA) quantified net benefit across a range of threshold probabilities to assess clinical utility. *p* < 0.05 was considered statistically significant.

## Results

### Baseline patient characteristics

In this study, a total of 17,768 participants were included, with 1,673 individuals in the GS group and 16,095 in the Non-GS group. The baseline characteristics of the two groups are presented below. GS group were significantly higher than Non-GS group in age, hip, waist, BMI, LDL, IBIL, TBA, ALT, TP, DB, CHO, TBIL, TG, SCC, LDH, and ALP, significant lower in sleep, AST/ALT. Moreover, there was significant differences between GS and Non-GS regarding the Sex, Education, Marriage, Insurance, Diabetes, HBP, Exercise, Smoke, Passive smoke, Drink, Taste, Frequency of consuming sweetmeat, FRY, Frequency of consuming nutrition, and Frequency of consuming tea. The result as detailed presented in Table 1.

**Table 1:** Baseline characteristics of the study subjects [X̄±*s*, *N*(%)].

Variable	Non-GS	GS	*t/χ* ^2^	*P/F*		Non-GS	GS	*t/χ* ^2^	*P/F*
Age (years)	54.97 ± 12.24	59.3 ± 10.72	−13.930	**<0.001**	HBP			22.163	**<0.001**
Hip (cm)	93.48 ± 6.69	94.43 ± 7.14	−5.507	**<0.001**	Yes	4,110 (25.54)	516 (30.84)		
Waist (cm)	80.1 ± 9.21	82.01 ± 9.28	−8.028	**<0.001**	No	11,985 (74.46)	1,157 (69.16)		
Pulse (bpm)	78.43 ± 11.67	78.5 ± 11.67	−0.238	0.812	Exercise			12.63	**<0.001**
Sleep (hours)	6.72 ± 1.41	6.61 ± 1.43	3.115	**0.002**	Yes	4,977 (30.77)	447 (26.67)		
BMI (kg/m^2^)	24.28 ± 3.22	24.98 ± 3.4	−8.370	**<0.001**	No	11,118 (69.23)	1,226 (73.33)		
LDL (mmol/L)	2.93 ± 0.74	3 ± 0.75	−3.303	**0.001**	Smoke			50.171	**<0.001**
IBIL (μmol/L)	12.96 ± 4.93	13.31 ± 4.83	−2.745	**0.006**	No	13,757 (85.47)	1,333 (79.68)		
TBA (μmol/L)	3.59 ± 5.23	6 ± 7.21	−17.227	**<0.001**	Yes	2,338 (14.53)	340 (20.32)		
ALT (U/L)	25.77 ± 19.91	27.65 ± 21.12	−3.655	**<0.001**	Passive smoke			13.661	**0.003**
TP (g/L)	77.64 ± 5.58	78.53 ± 5.98	−6.152	**<0.001**	No	12,258 (76.16)	1,210 (72.33)		
DB (μmol/L)	3.99 ± 1.88	4.12 ± 1.94	−2.808	**0.005**	1–2 times	1,210 (7.52)	137 (8.19)		
CHO (mmol/L)	5.23 ± 1	5.33 ± 1.02	−3.872	**<0.001**	3–5 times	571 (3.55)	77 (4.60)		
TBIL (μmol/L)	16.95 ± 6.39	17.43 ± 6.34	−2.946	**0.003**	>5 times	2,056 (12.77)	249 (14.88)		
POT (mmol/L)	4.09 ± 0.33	4.08 ± 0.33	0.799	0.424	Drink			8.1	**0.017**
ASP (U/L)	26.73 ± 12.88	27.37 ± 12.49	−1.927	0.054	No	1,1,517 (71.56)	1,147 (68.56)		
TG (mmol/L)	1.66 ± 1.24	1.91 ± 1.46	−7.891	**<0.001**	Yes	4,020 (24.98)	485 (28.15)		
HDL (mmol/L)	1.81 ± 0.48	1.82 ± 0.48	−0.513	0.608	Quit	558 (3.47)	61 (3.29)		
AST/ALT (U/L)	1.22 ± 0.46	1.16 ± 0.44	4.887	**<0.001**	Drink water				0.149
SCC (ng/mL)	0.75 ± 0.19	0.77 ± 0.19	−4.574	**<0.001**	Tap	7,165 (44.52)	744 (44.47)		
ALB (g/L)	47.4 ± 2.81	47.35 ± 2.74	0.741	0.459	Purified	8,672 (53.88)	901 (53.86)		
LDH (U/L)	187.78 ± 35.54	190.49 ± 33.38	−2.984	**0.003**	Mineral	248 (1.54)	24 (1.43)		
ALP (U/L)	83.91 ± 26.42	86.9 ± 26.63	−4.403	**<0.001**	Other	10 (0.06)	4 (0.24)		
Sex			26.988	**<0.001**	Oil			1.823	0.402
Male	5,578 (34.73)	474 (28.33)			Vegetable	1,5,116 (93.92)	1,584 (94.68)		
Female	10,517 (65.34)	1,199 (71.67)			Animal	719 (4.47)	63 (3.77)		
Education			18.415	**<0.001**	Other	260 (1.62)	26 (1.55)		
Primary	5,703 (35.43)	637 (38.08)			Taste			42.54	**<0.001**
Middle	5,777 (35.89)	601 (35.92)			Light	9,281 (57.66)	903 (53.97)		
High	2,715 (16.4)	287 (17.15)			Moderation	2,720 (16.90)	277 (16.56)		
Bachelor	1,976 (12.28)	148 (8.85)			Spicy	3,321 (20.63)	352 (21.04)		
Marriage				**<0.001**	Greasy	773 (4.80)	141 (8.43)		
Yes	14,473 (89.92)	1,482 (88.58)			Espeed			0.976	0.614
No	245 (1.52)	3 (0.18)			Slow	1,072 (6.66)	119 (7.11)		
Divorce	402 (2.50)	45 (2.69)			Moderate	9,136 (56.76)	931 (55.65)		
Separation	630 (3.91)	60 (3.59)			Fast	5,887 (36.58)	623 (37.24)		
Widowed	345 (2.14)	83 (4.96)			Sweetmeat			9.846	**0.02**
Insurance			22.731	**<0.001**	NO	6,954 (43.21)	737 (44.05)		
No	359 (2.15)	31 (1.8)			1–2 times	7,368 (45.78)	715 (42.74)		
Urban	9,983 (59.82)	1,129 (65.6)			3–5 times	1,386 (8.61)	172 (10.28)		
Rural	5,883 (35.25)	514 (29.87)			>5 times	387 (2.40)	49 (2.93)		
Commercial	174 (1.04)	19 (1.1)			Fry			115.94	<0.001
Other	290 (1.74)	28 (1.63)			No	9,028 (56.09)	856 (51.17)		
COPD			0.426	0.808	1–2 times	6,712 (41.70)	710 (42.44)		
No	15,465 (96.09)	1,603 (95.82)			3–5 times	326 (2.03)	92 (5.5)		
Uncertain	515 (3.20)	56 (3.35)			>5 times	29 (0.18)	15 (0.90)		
Yes	115 (0.71)	14 (0.81)			Coffee			6.547	0.088
Tumor				0.74	No	14,648 (91.01)	1,548 (92.53)		
No	14,050 (87.29)	1,445 (86.37)			1–2 times	1,232 (7.65)	113 (6.75)		
Benign	1,767 (10.98)	196 (11.72)			3–5 times	123 (0.76)	7 (0.42)		
Malgnant	167 (1.04)	21 (1.26)			>5 times	92 (0.57)	5 (0.30)		
Both	10 (0.06)	1 (0.06)			Nutrient			60.339	<0.001
Uncertain	101 (0.63)	10 (0.60)			Regular intake	5,255 (32.65)	409 (24.45)		
Diabetes			128.19	**<0.001**	Irregular intake	8,614 (53.52)	951 (56.84)		
No	14,864 (92.35)	1,412 (84.40)			No	2,226 (13.83)	313 (18.71)		
Yes	1,173 (7.29)	254 (15.18)			Tea			13.795	0.003
Other	60 (0.36)	7 (0.42)			No	9,026 (56.08)	1,017 (60.79)		
					1–2 times	2,887 (17.94)	267 (15.96)		
					3–5 times	988 (6.14)	95 (5.68)		
					>5 times	3,194 (19.84)	294 (17.57)		

All the bold P/F means there was a statistically significant difference between the two groups.

### Variable selection

**Figure 2:**
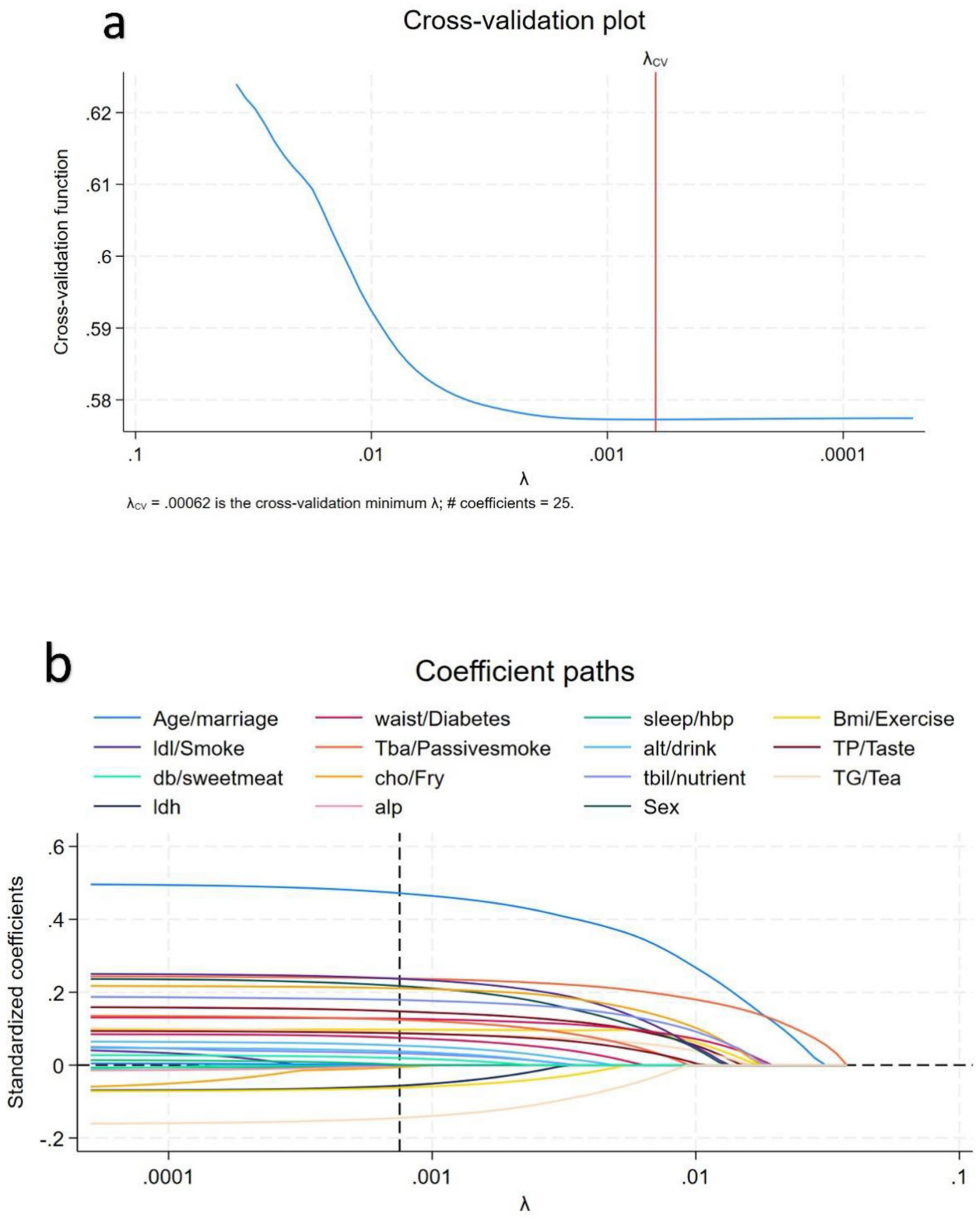
LASSO regression for variable selection. **(a)** is for Lasso Cross-Validation Plot, **(b)** is for Lasso Coefficient Path Plot. HBP means high blood pressure, LDL means low density lipoprotein, TBA means total bile acid, alt means alanine aminotransferase, TP means total protein, CHO means cholesterol, TBIL means total bilirubin, TG means triglyceride, LDH means lactate dehydrogenase, ALP means alkaline phosphatase.

Subsequently, LASSO regression was employed to screen the mentioned 17 statistically significant variables. Cross-validation (Figure 2a) determined the optimal penalty parameter *λ* = 0.00062, under which a total of 25 variables were selected and incorporated into the model. The coefficient path plot (Figure 2b) showed that as the penalty intensity increased, the coefficients of some variables were shrunk to zero. At the optimal *λ* value, 25 variables, including age, marital status, waist circumference, diabetes, sleep/HBP, BMI, physical activity, blood lipids, smoking, retained non-zero coefficients, suggesting their independent associations with the target outcome.

### Logistic regression and random forest model construction

The selected variables were incorporated into a multivariate logistic regression model. The results demonstrated that age, waist, irregular nutrient intake, BMI, smoking, ALT, TBA, TP, TG, diabetes, sex, drink, FRY, greasy diets and passive smoking were identified as risk factors for gallstone (OR > 1, *p* < 0.05). In contrast, tea intake and regular exercise were found to be protective factors (OR > 1, *p* < 0.05). Furthermore, variance inflation factor (VIF) analysis indicated that values of all variables with average VIF 1.16, which less than 10, suggesting no multicollinearity among the included variables (Table 2).

**Table 2:** Multiple variables logistic regression analysis of gallstone.

Variable	*β*	OR	*z*	*p > z*	95% CI
Age	0.039	1.040	11.85	<0.001	[1.032, 1.046]
Waist	0.017	1.018	4.78	<0.001	[1.011,1.024]
TBA	0.041	1.042	8.66	<0.001	[1.032, 1.051]
ALT	0.004	1.004	2.84	0.004	[1.001, 1.006]
TP	0.022	1.022	3.56	<0.001	[1.009, 1.034]
TG	0.066	1.068	3.16	0.002	[1.025, 1.113]
LDH	−0.002	0.998	−1.68	0.093	[0.996, 1.001]
Tea					
1–2 times	−0.036	0.965	−0.4	0.688	[0.811, 1.147]
3–5 times	−0.322	0.725	−2.24	0.025	[0.546, 0.96]
>5 times	−0.437	0.646	−4.74	<0.001	[0.538, 0.773]
Diabetes					
Yes	0.493	1.637	5.11	<0.001	[1.354, 1.977]
Prediabetes	−0.238	0.788	−0.43	0.666	[0.267, 2.321]
Passive smoke					
1–2 times	0.308	1.361	2.58	0.010	[1.077, 1.719]
3–5 times	0.529	1.697	3.46	0.001	[1.257, 2.29]
>5 times	0.365	1.440	3.86	<0.001	[1.196, 1.733]
Fry					
1–2 times	0.247	1.280	3.68	<0.001	[1.122, 1.46]
3–5 times	1.186	3.273	7.61	<0.001	[2.412, 4.442]
>5 times	1.942	6.970	5.39	<0.001	[3.441, 14.118]
Smoke					
Yes	0.820	2.271	9.24	<0.001	[1.907, 2.702]
Exercise					
Yes	−0.210	0.811	−2.89	0.004	[0.703, 0.934]
Taste					
Moderation	−0.032	0.969	−0.34	0.731	[0.809, 1.159]
Spicy	0.064	1.066	0.78	0.436	[0.907, 1.253]
Greasy	0.536	1.709	4.2	<0.001	[1.331, 2.194]
Sex					
Female	0.499	1.647	6.55	<0.001	[1.418, 1.912]
Nutrient					
Irregular intake	0.499	1.647	5.73	<0.001	[1.388, 1.953]
NO	0.700	2.014	6.86	<0.001	[1.649, 2.46]

*β*, partial regression coefficient; OR, odds ratio; CI, confidence interval.

**Figure 3:**
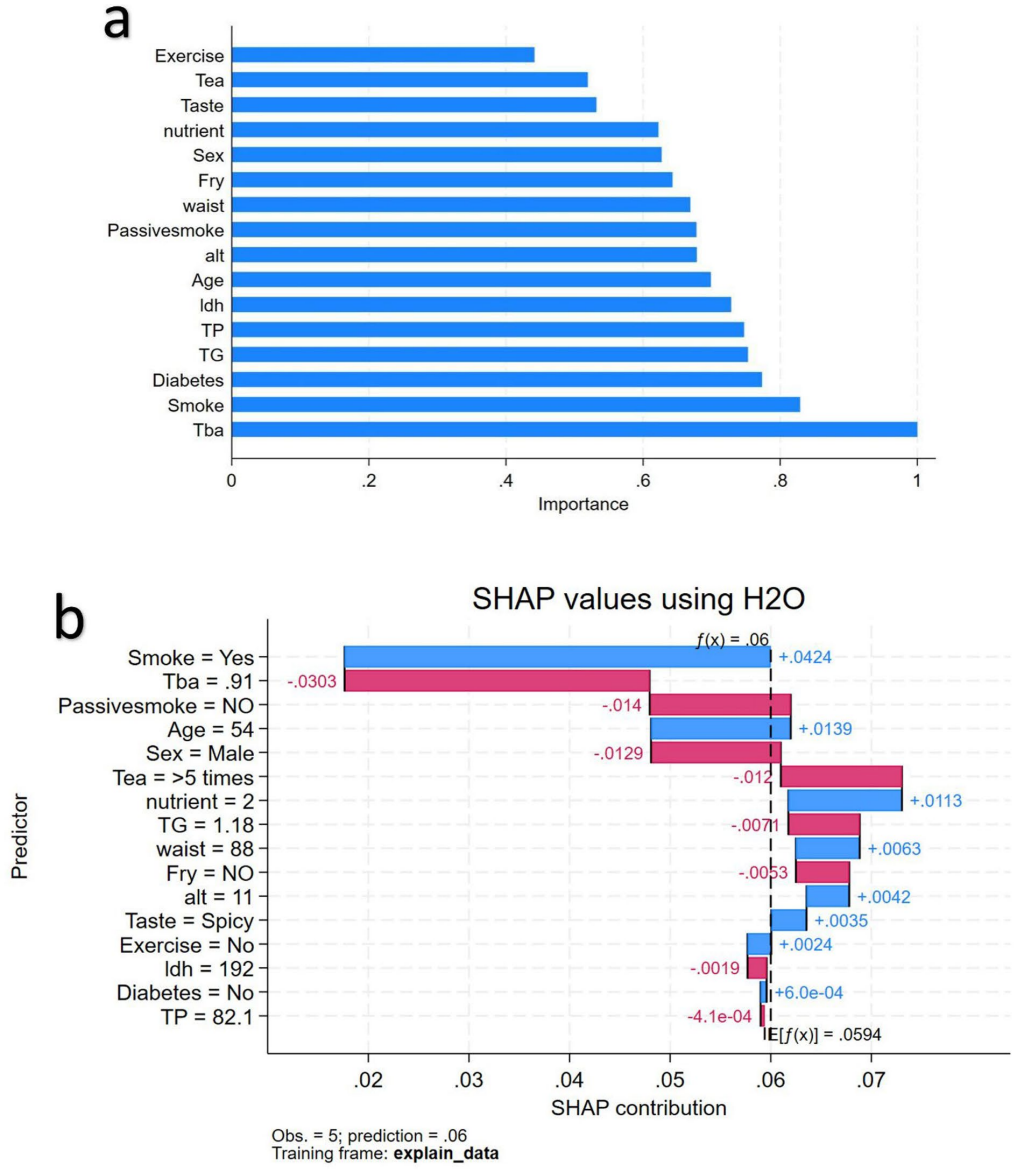
Shap analysis of random forest model. **(a)** is Shap Bar Plot, **(b)** is shap value plot.

**Figure 4:**
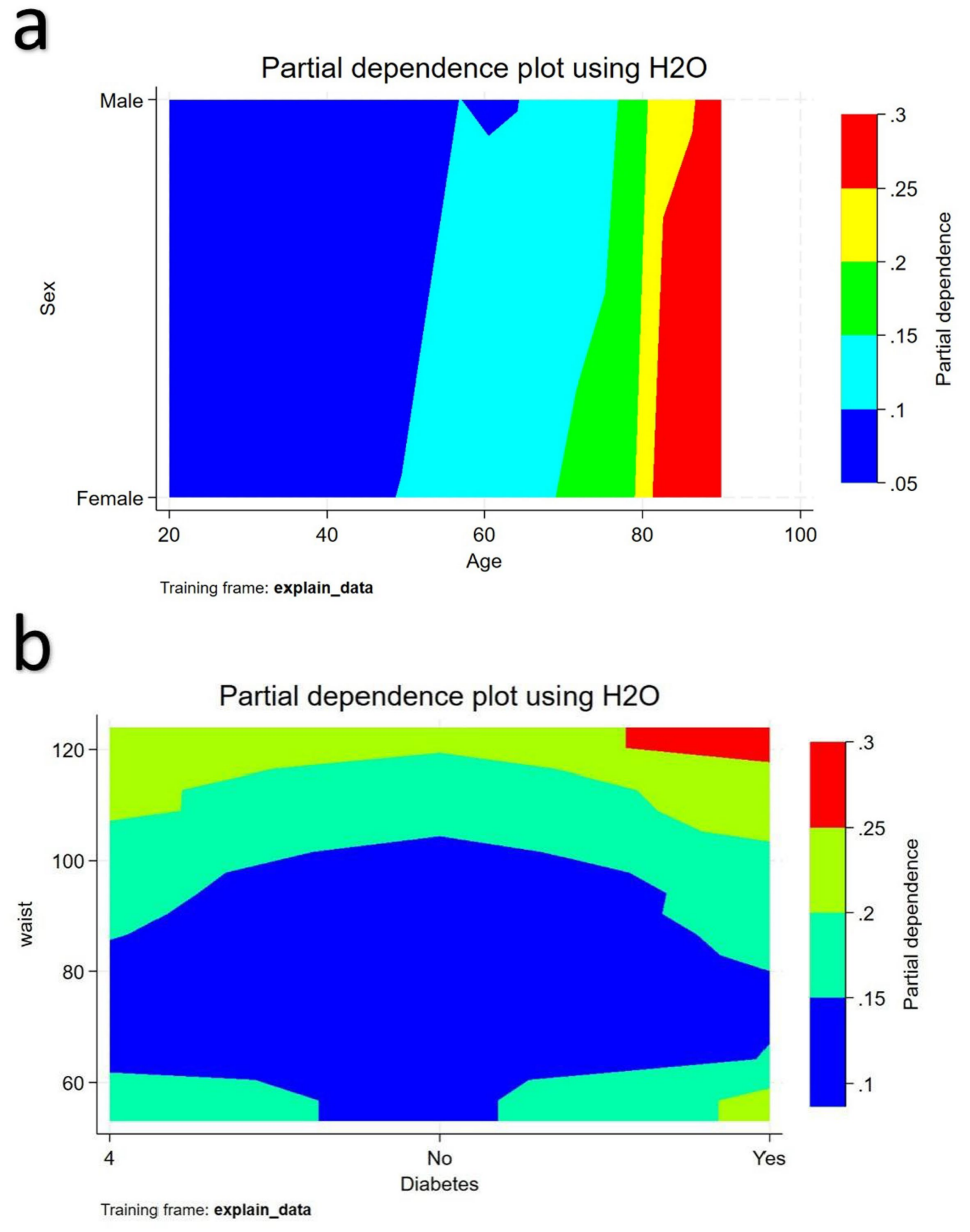
Shap partial dependence plot of random Forest model. **(a)** is for interaction between age and sex, **(b)** is for interaction between diabetes and waist.

A random forest algorithm was further employed to construct a predictive model, and model interpretability analysis was conducted to reveal variable importance and underlying mechanisms. The ranking of variable importance showed that TBA, smoking status, diabetes, TG, and TP were the top five variables contributing the most to the model (Figure 3a). The SHAP waterfall plot further clarified the predictive drivers for individual samples, intuitively presenting the direction and magnitude of each variable’s contribution to the individual outcome prediction (Figure 3b). PDP revealed interactive effects among key variables: age and sex exhibited a synergistic effect, with elderly males showing a significantly higher risk of the outcome; waist circumference and diabetes status also displayed an interactive effect, where individuals with high waist circumference and concurrent diabetes had a significantly higher risk of the outcome (Figure 4).

### Performance indicators comparison

The performance metrics of both models are compared in Table 3. The random forest model exhibited higher specificity (0.91) than logistic regression model (0.81), while its sensitivity (0.86) was slightly lower (0.93). The positive likelihood ratio (LR+) was substantially higher for the random forest (9.65 vs. 4.91), and the negative likelihood ratio (LR–) was comparable (0.15 vs. 0.12). Moreover, the random forest model achieved a lower Brier score (0.073 vs. 0.075) and a higher F1 score (63.41% vs. 48.58%). These results indicate that the random forest model provides a superior balance between sensitivity and specificity, reduces false positives, and offers improved overall predictive accuracy.

**Table 3:** Performance indicators and calibration assessment of two models.

Model	AUC	Sen	Spe	LR^+^	LR^−^	Brier	F1
Random forest	0.747	0.86	0.91	9.65	0.15	0.073	63.41%
Logistic	0.694	0.93	0.81	4.91	0.12	0.075	48.58%

AUC, area under curve; Sen, sensitivity; Spe, specificity; LR^+^, positive likelihood ratio; LR^−^, negative likelihood ratio; Brier, Brier score; F1, F1 score.

### Model performance evaluation and calibration

**Figure 5:**
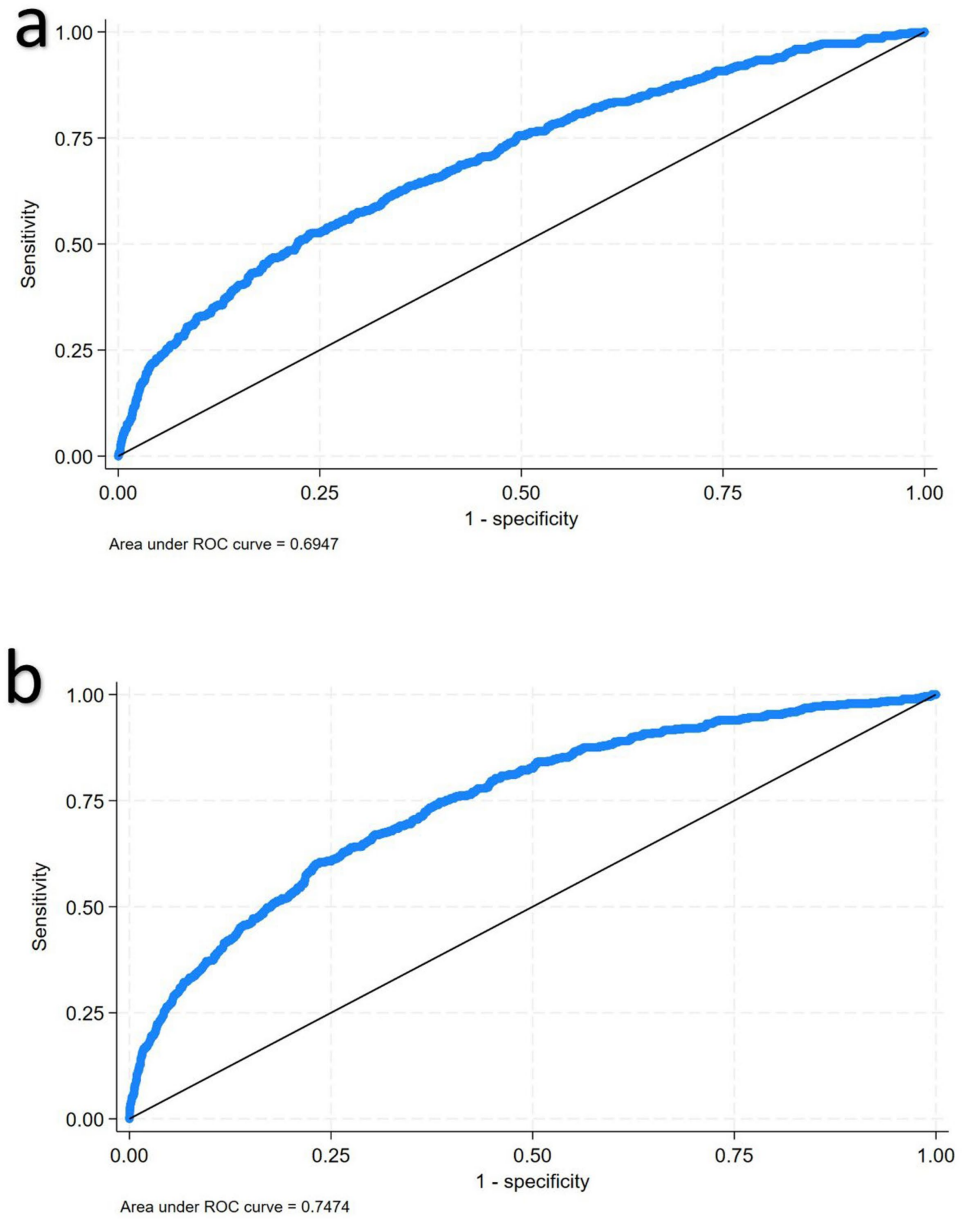
ROC curves for the prediction model. **(a)** is for Logistic regression model, **(b)** is for random forest model.

**Figure 6:**
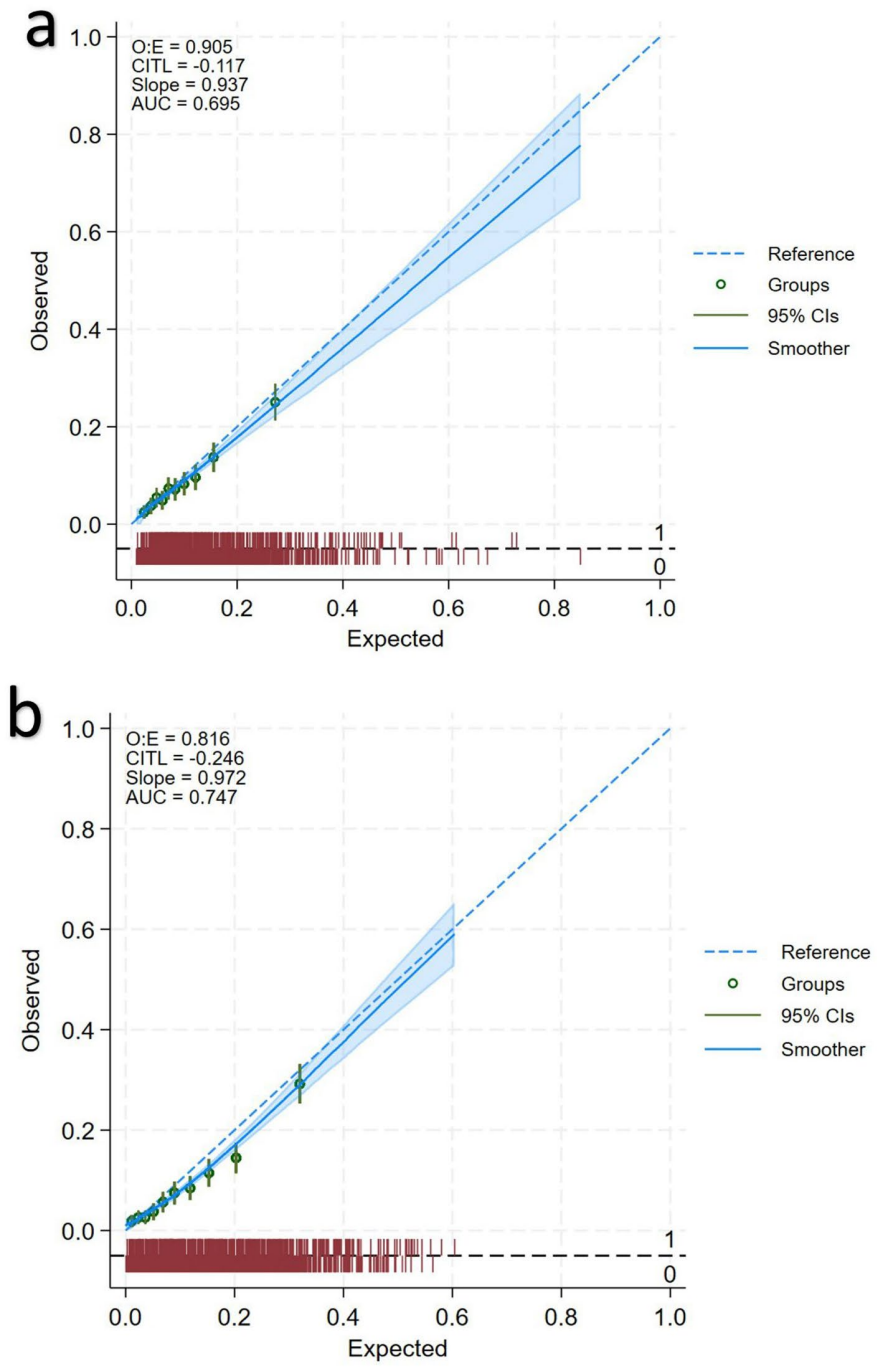
Calibration plot for two prediction model. **(a)** is for Logistic regression model, **(b)** is for random forest model. O:E, observed-to-expected ratio, CITL, calibration-in-the-large, Slope, calibration slope.

The discriminative ability of the predictive model was evaluated using ROC curves. The logistic regression model achieved an AUC of 0.694 (95% *CI*: 0.669–0.720), while random forest model achieved an AUC of 0.747 (95% *CI*: 0.724–0.771), see Figure 5. Additionally, calibration curves were employed to assess the consistency between two different models The calibration curves for the logistic regression model and random forest model all closely aligned with the reference line, suggesting good concordance between predicted and actual probabilities The Hosmer–Lemeshow test result shows that *χ*^2^ = 8.21, *p* = 0.413, indicating that the model is well calibrated. (Figure 6, Table 3).

### Clinical efficacy evaluation

**Figure 7:**
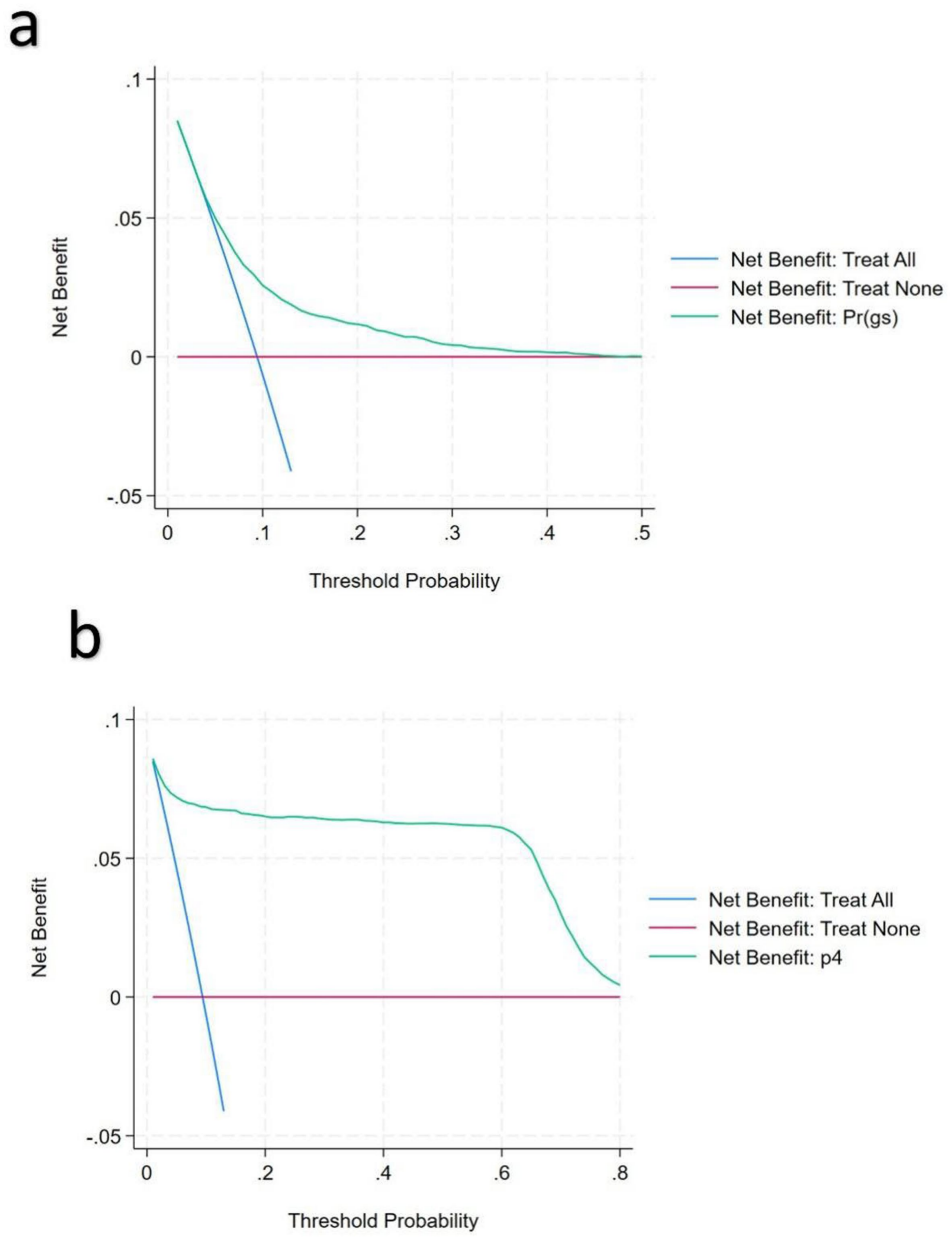
Decision curve analysis of the prediction model. **(a)** is for Logistic regression model, **(b)** is for random forest model.

The DCA demonstrated that the net benefits of both models for treatment decision-making were higher than the strategies of treating all patients or treating none. The blue curve represents the “treat all” strategy, while the red curve indicates the “treat none” strategy, which yields zero net benefit across the entire range. Moreover, these results indicate that the random forest model provides substantial net benefit and reliable predictive accuracy, supporting its use for risk prediction of GS (Figure 7).

## Discussion

We developed a risk factor identification model for gallstone disease in southwestern China by screening a wide range of relevant variables. The model was validated and its clinical utility assessed, demonstrating good predictive performance. It effectively identifies individuals at risk of GS disease, providing a useful tool for risk detection.

Gallstone disease is a common biliary disorder, affecting approximately 10% of the Chinese population. Due to the country’s large population, this represents a considerable public health burden. Our study identified smoking and alcohol consumption as significant risk factors for gallstone. Previous research has shown that smoking can lead to abnormal lipid metabolism, elevated cholesterol and LDL levels, reduced hepatic bile acid excretion, and increased risk of gallstone formation (13). Both smoking and alcohol intake may delay gastric and gallbladder emptying, and chronic alcohol use can raise serum TG levels, adversely affecting systemic metabolism (3). Moreover, our study demonstrated the impact of passive smoking on gallstone disease. Individuals regularly exposed to secondhand smoke had a 1.4 times higher risk than those without exposure. Although the mechanism is not fully understood, it has been confirmed that several carcinogenic chemicals in passive smoke (such as 4-aminobiphenyl, benzene, and nickel compounds) are presented with significantly higher concentrations than in actively inhaled smoke (14, 15), which were the main substance affects cancer.

There was a higher incidence in women than in men. This difference is mainly attributable to the lipophilic nature of estrogen, which allows it to passively enter cells and function as a transcription factor. In hepatocytes, estrogen enhances cholesterol secretion, leading to increased cholesterol saturation in bile (16). High waist circumference is closely associated with gallstone. Fat accumulation disrupts hepatic lipid metabolism, raising biliary cholesterol levels. When cholesterol concentration exceeds the solubilizing capacity of bile acids and phospholipids, crystals form, eventually promoting gallstone development (17). Excess body weight may also impair metabolic function which increased the risk of gallstone (18).

Dietary habits and lifestyle also affect gallstone formation. Our study identified regular exercise and nutritional supplementation as protective factors of gallstone. Regular exercise was defined as engaging in at least 150 min of moderate-intensity aerobic physical activity per week. Increased physical activity helps prevent gallstones by raising HDL and lowering TG levels (19). Irregular nutrient intake means the specific emphasis on the frequency and regularity of such intake. Vitamin C deficiency has been shown to impair cholesterol 7α-hydroxylation, which reducing bile acid synthesis and leading to cholesterol supersaturation in bile (20). Thus, extra vitamin C supplementation may help lower gallstone risk. Niacin supplementation has also been found to raise HDL and lower LDL cholesterol (21). Our study further revealed a strong association between long-term fried food consumption and gallstone disease. In our study, FRY intake was assessed by asking participants how many times per week they consumed fried foods. High fat intake is a common clinical factor of acute gallstone attacks. Fried foods are rich in saturated fats, which are poorly metabolized. Long-term excessive intake of fat will stimulates gallbladder bile secretion and increases bile viscosity, accelerating cholesterol crystallization and deposition (22).

Multiple studies have confirmed that TBA is closely correlated with gallstone development (23). Cholesterol supersaturation in bile (resulting from abnormalities in TBA synthesis and transport) is the primary cause of gallstone formation (24). Clinical studies have shown that impaired function of organic solute transporters OSTα and OSTβ, contributes to gallstone formation in non-obese patients with glycogen storage disease (25). Additionally, elevated serum albumin levels may indicate systemic oxidative stress, which is an important mechanism in gallstone pathogenesis. Oxidative stress promotes reactive oxygen species (ROS) production (26). The interaction between ROS and biliary cholesterol accelerates crystal formation, thereby facilitating gallstone development (27).

Random forest model achieved a moderate AUC of 0.747, a common performance level for multifactorial diseases, limited by the condition’s etiological complexity, asymptomatic disease misclassification, and self-reported lifestyle variable measurement error. SHAP analysis identified TBA, smoking status, diabetes, TG and TP as the top predictive factors, consistent with known pathophysiologys (4). Unlike logistic regression model, the random forest model captured critical non-linear interactions, including accelerated gallstone risk in older males and synergistic risk between central obesity and diabetes. Clinically, the model’s high specificity leads to a 9% false positive rate, which is largely acceptable given the non-invasive nature of follow-up ultrasound, while its 86% sensitivity implies some missed opportunities for intervention, potentially leading to delayed complications and more costly treatments (12).

Although the random forest model showed moderate discriminative ability, DCA revealed that it offered superior clinical utility compared with logistic regression. In contrast to the logistic model, which outperformed the reference strategies only within a narrow threshold range, the random forest maintained a positive net benefit across a broad spectrum of clinically relevant thresholds (0 ~ 0.8). This advantage likely arises from the model’s capacity to capture non-linear relationships and interactions among predictors, thereby enabling more subtle risk stratification and potentially reducing unnecessary interventions (28, 29).

Our study found an interesting result: although no significant difference in coffee consumption was observed between the two groups, regular tea intake per week was identified as a protective factor against gallstone formation. Both coffee and tea have been previously proved to a reduced risk of gallstones (30), the reason may due to caffeine’s ability to inhibit gallbladder absorption and prevent cholesterol crystallization (31). We speculate that the absence of a coffee-related protective effect in our study may be related to the age of the research population. Coffee is far less common than tea among middle-aged and older adults in China, which may explain why tea drinkers showed a lower risk of gallstone disease.

### Limitations

Compared to previous studies, the risk factor identification model developed in our study incorporated a broader range of variables. Model validation demonstrated that the model has good predictive performance. However, as a cross-sectional study, it cannot fully elucidate the causal relationships between the variables and the disease. Besides, self-reported lifestyle information (smoking, alcohol, diet, physical activity) may suffer from recall and social desirability bias,which could affect the accuracy of these variables. The study population consisted of individuals who voluntarily participated in health screenings, may have higher health awareness than the general population. Moreover, although TBA was the most important variable in our model, its non-routine use in primary care and community screening limits generalizability. Nevertheless, our findings remain valuable for regions with high gallstone prevalence. Future research could develop a simplified TBA-free model for broader application.

Additionally, external dataset and prospective studies are warranted in the future to further validate and enhance the predictive power of the model.

## Data Availability

The raw data supporting the conclusions of this article will be made available by the authors upon reasonable request.
